# Will emergency and surgical patients participate in and complete alcohol interventions? A systematic review

**DOI:** 10.1186/1471-2482-11-26

**Published:** 2011-09-23

**Authors:** Bolette Pedersen, Kristian Oppedal, Lisa Egund, Hanne Tønnesen

**Affiliations:** 1 WHO Collaborating Centre for Evidence-based Health Promotion in Hospitals and Health Services, Bispebjerg University Hospital, Copenhagen NV, Denmark; 2 Clinical Alcohol Research, Faculty of Medicine, Lund University, Sweden; 3 Alcohol and Drug Research Western Norway, Stavanger University Hospital, Stavanger, Research Unit for General Practice, Uni Health, Bergen, Norway; 4 Orthopaedic Department, Skane University Hospital Malmö, Lund University, Sweden

## Abstract

**Background:**

In the everyday surgical life, staff may experience that patients with Alcohol Use Disorders (AUDs) seem reluctant to participate in alcohol intervention programs. The objective was therefore to assess acceptance of screening and intervention as well as adherence to the intervention program among emergency department (ED) and surgical patients with AUDs.

**Methods:**

A systematic literature search was followed by extraction of acceptance and adherence rates in ED and surgical patients. Numbers needed to screen (NNS) were calculated. Subgroup analyses were carried out based on different study characteristics.

**Results:**

The literature search revealed 33 relevant studies. Of these, 31 were randomized trials, 28 were conducted in EDs and 31 evaluated the effect of brief alcohol intervention. Follow-up was mainly conducted after six and/or twelve months.

Four in five ED patients accepted alcohol screening and two in three accepted participation in intervention. In surgical patients, two in three accepted screening and the intervention acceptance rate was almost 100%. The adherence rate was above 60% for up to twelve months in both ED and surgical patients. The NNS to identify one eligible AUD patient and to get one eligible patient to accept participation in alcohol intervention varied from a few up to 70 patients.

The rates did not differ between randomized and non-randomized trials, brief and intensive interventions or validated and self-reported alcohol consumption. Adherence rates were not affected by patients' group allocation and type of follow-up.

**Conclusions:**

Most emergency and surgical patients with AUD accept participation in alcohol screening and interventions and complete the intervention program.

## Background

Staff working in emergency or surgical departments will frequently encounter patients with alcohol use disorders (AUDs). These include hazardous drinking exceeding a weekly or daily threshold as well as harmful and dependent drinking [1]. In emergency departments (ED) up to four in ten patients suffer from AUDs [2,3], and AUDs are especially widespread among trauma patients [4]. The frequency of AUD patients in elective surgery varies according to diagnosis and type of operation; for example are less than one in ten women undergoing hysterectomy AUD patients [5]. On the contrary, in patients undergoing tumor resection of the upper digestive tract up to six in ten suffer from AUDs [6]. Several screening tools have been developed and tested to detect AUDs including CAGE ('Cut down, Annoyed, Guilty, Eye-opener') [7], MAST (Michigan Alcoholism Screening Test) [8], and AUDIT (Alcohol Use Disorder Identification Test) [9].

Patients with AUDs develop more complications following surgery and are more often readmitted to EDs [10,11]. Some alcohol intervention programs have proven a positive effect on these outcomes through a reduction of alcohol consumption or abstinence [12,13], but overall the evidence for alcohol intervention programs in hospitals is unclear [14-16]. Furthermore, staff may expect or experience difficulties with getting ED or surgical patients to participate in alcohol intervention programs [17,18].

This review will therefore assess acceptance of alcohol screening, acceptance of alcohol intervention and adherence to intervention among AUD patients in emergency and surgical departments, as knowledge about these rates are important when planning future alcohol interventions.

## Methods

### Criteria for considering studies for this review

#### Types of studies

Only randomized and controlled clinical trials were included. Studies including consecutive adult elective or acute patients with AUDs treated in a surgical or emergency department were considered. Reviews and other types of secondary literature were excluded. Other exclusion criteria were population or staff interventions and studies conducted in other settings than hospitals.

#### Types of data

Papers and abstracts were included if they provided data on definition of AUDs and identification method, numbers/rates regarding acceptance and adherence, and type of alcohol intervention.

#### Types of methods

Interventions of interest were all alcohol intervention programs focusing on alcohol reduction or cessation. Interventions could be brief or intensive, including programs with pharmaceutical interventions for alcohol withdrawal and relapse prophylaxis. Control groups were defined as assessment of AUDs only or treatment as usual.

### Types of outcome measures

Screening acceptance rate = Number screened for AUDs/Total patient population;

Intervention acceptance rate = Number participating in intervention/Number of eligible AUD patients fulfilling study inclusion criteria;

Adherence rates = Number of patients at follow-up(s)/Number of patients accepting intervention.

Numbers needed to screen (NNS) = 1/(number of eligible/total patient population) and 1/(number accepting intervention/total patient population).

### Electronic searches and other resources

The literature search was performed in the following databases: MEDLINE, the Cochrane Central Register of Controlled Trials (CENTRAL), EMBASE and Cinahl (see detailed search strategy for each database in table 1). No time or language restrictions were set. Both full paper articles as well as abstracts were considered. Titles and abstracts were screened to exclude any clearly irrelevant papers. All potentially relevant papers and abstract were then assessed in accordance with the inclusion and exclusion criteria. Two authors (BP and KO) were responsible for screening and assessment of abstracts and full paper articles.

**Table 1:** Search strategy for the electronic databases

MEDLINE search strategy 1. "Alcohol-Related Disorders"[Mesh] OR "Alcohol Drinking"[Mesh] OR "Alcoholism"[Mesh] OR alcohol abuse OR alcohol use OR alcohol disorder OR alcohol consumption OR alcohol intake OR alcohol behaviour OR hazardous drinking OR harmful drinking OR alcohol dependence OR risky drinking 2. "Temperance"[Mesh] OR alcohol intervention OR alcohol education OR alcohol program OR alcohol brief intervention OR alcohol reduction OR alcohol cessation OR alcohol withdrawal OR alcohol abstinence 3. "Emergency Service, Hospital"[Mesh] OR "Wounds and Injuries"[Mesh] OR "Surgical Procedures, Operative"[Mesh] OR surgical treatment OR surgical patient OR trauma treatment OR trauma patient OR emergency treatment OR emergency patient 4. #1 AND #2 AND #3 (limits: Randomized controlled trial, controlled clinical trial; adults)	The Cochrane Central Register of Controlled Trials (CENTRAL) search strategy 1. "Alcohol-Related Disorders"[Mesh] OR "Alcohol Drinking"[Mesh] OR "Alcoholism"[Mesh] OR alcohol abuse OR alcohol use OR alcohol disorder OR alcohol consumption OR alcohol intake OR alcohol behaviour OR hazardous drinking OR harmful drinking OR alcohol dependence OR risky drinking 2. "Temperance"[Mesh] OR alcohol intervention OR alcohol education OR alcohol program OR alcohol brief intervention OR alcohol reduction OR alcohol cessation OR alcohol withdrawal OR alcohol abstinence 3. "Emergency Service, Hospital"[Mesh] OR "Wounds and Injuries"[Mesh] OR "Surgical Procedures, Operative"[Mesh] OR surgical treatment OR surgical patient OR trauma treatment OR trauma patient OR emergency treatment OR emergency patient 4. #1 AND #2 AND #3
**EMBASE search strategy** 1. exp alcohol abuse/OR alcohol consumption/OR drinking behaviour/OR alcoholism/ 2. exp alcohol abstinence/OR alcohol withdrawal/OR patient counselling/ 3. exp emergency health service/OR emergency care/OR emergency surgery/OR emergency patient/OR emergency ward/OR emergency treatment/OR surgical ward/OR surgical patient/OR injury/OR surgery/ 4. exp intervention study/OR randomised controlled trial/OR controlled clinical trial/OR randomisation/OR clinical trial 5. #1 AND #2 AND #3 AND #4	**Cinahl search strategy** 1. Alcohol-Related Disorder+ OR alcohol Drinking OR alcoholism OR alcohol abuse OR alcohol use OR alcohol disorder OR alcohol consumption OR alcohol intake OR alcohol behaviour OR hazardous drinking OR harmful drinking OR alcohol dependence 2. Temperance OR alcohol intervention OR alcohol education OR alcohol program OR brief intervention OR reduction OR cessation OR withdrawal OR abstinence 3. Emergency health service OR emergency care OR emergency surgery OR emergency patient OR emergency ward OR emergency treatment OR surgical ward OR surgical patient OR injury OR surgery 4. #1 AND #2 AND #3

Search conducted 17^th ^of March 2010, updated 25^th ^of November 2010

Reference lists and related articles from the included papers were hand-searched to identify other relevant studies. Additional database searches were conducted in http://www.controlled-trials.com, http://www.clinicaltrials.gov and http://www.centerwatch.com. A third author (LE) was responsible for this part. Any disagreement was solved by consensus involving all authors (BP, KO, LE and HT).

### Data abstraction

The following information was extracted: Type of patients, definition and method(s) used to identify AUDs, total patient population, number screened for AUDs, number of eligible AUD patients, number of eligible AUD patients accepting intervention, type of alcohol intervention, type and time for follow-up visit(s) and number of patients at follow-up.

### Data analysis

Acceptance and adherence rates as well as NNS were calculated for ED and surgical patients respectively. Results are given as median (range).

The following subgroup analyses were carried out comparing randomized clinical trials versus controlled clinical trials, intensive intervention programs versus brief intervention, bio-chemical validation of alcohol consumption versus self-reported consumption only, intervention versus control groups (follow-up rates only) and follow-up by attendance only versus follow-up by contact only (phone, e-mail etc.).

Chi-square tests were used to compare the differences in weighted proportions. A 5% significance level was accepted.

## Results

### Characteristics of included studies

**Figure 1:**
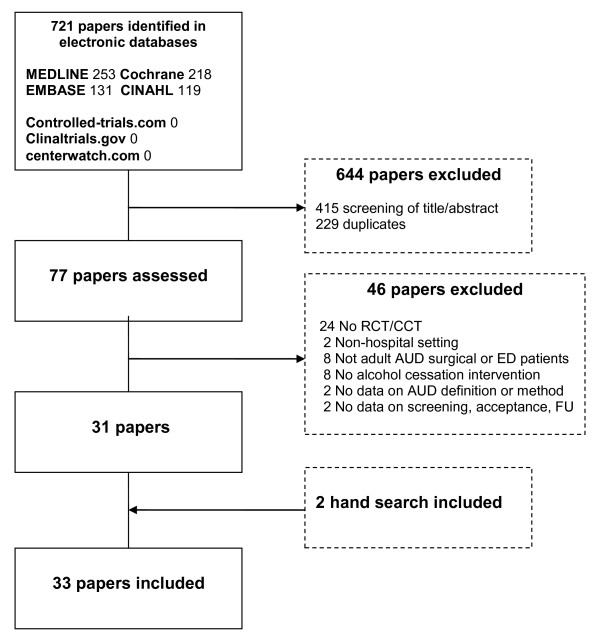
**Trial profile**.

Thirty-three papers were included in the review [11-13,19-48]; see trial profile in Figure 1. The studies originated from United States (16), United Kingdom (6), Spain (3), Australia (2), Sweden (2), Denmark (1), Finland (1), Germany (1) and Switzerland (1). The trials were published 1988 to 2010.

The majority of the studies (31) were randomized trials, 28 were conducted in EDs and 31 evaluated the effect of brief alcohol interventions. Type of follow-up varied between the studies; in 15 studies patients were contacted by phone and/or mail and in eight studies the patients had to attend follow-up. The remaining studies used a combination of the two or type of follow-up was not reported or performed. Follow-up was conducted after one month in two studies and after three months in twelve studies. Seventeen studies had follow-up after six and twelve months. Twenty studies included more than one follow-up visit. In 16 studies alcohol consumption was biochemically validated. Characteristics of the included studies are presented in table S2 (see additional file 1: Table S2 - Characteristics of 33 included studies involving ED or surgical patients).

### Acceptance and retention rates

#### Emergency department patients

**Figure 2:**
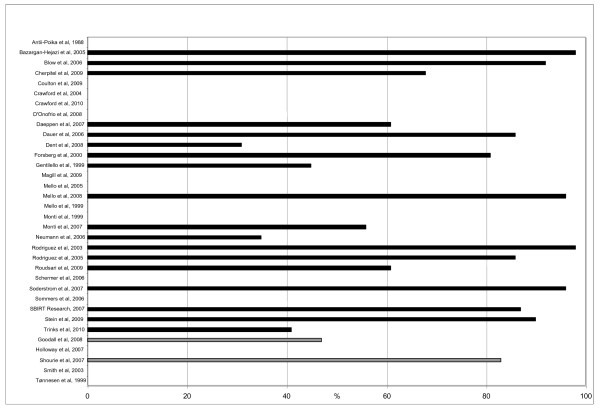
**Screening acceptance rate in 20 studies**. Black bars represent ED patients and grey bars surgical patients.

In 18 of 28 studies the total patient population was given, in which the median size was 5,640 ranging from 697 to 32,965 patients. Twenty-six studies reported the number of screened patients. Eighteen studies presented both numbers, and their screening acceptance rate was 83% in median (range 31-98%); see Figure 2.

**Figure 3:**
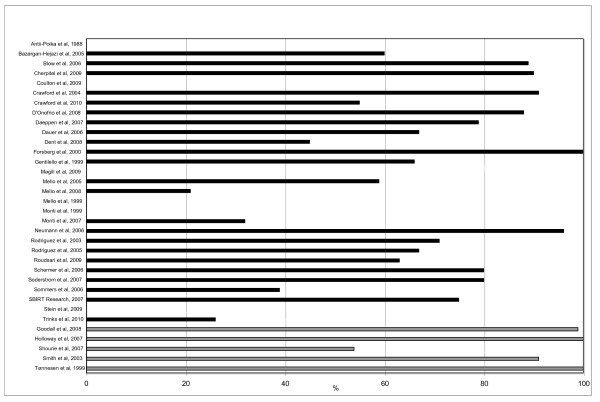
**Intervention acceptance rate in 28 studies**. Black bars represent ED patients and grey bars surgical patients.

The number of patients accepting intervention was reported in all 28 studies; however, not all had information on number of eligible AUD patients. In the 23 studies that reported both, the acceptance rate for intervention among the eligible patients was 67% (21-96%); see Figure 3.

All but one trial [42] conducted one or more follow-up visits. Only one study [28] had a one-month follow-up visit and the adherence rate was 62%. The adherence rate after three months was 67% (54-96%) based on ten studies [19,20,22,27,28,35,36,39,40,48], after six months 72% (45-89%) based on 15 studies [11,23-25,27,29,30,33,36-38,41,44,47,48] and 67% (27-92%) after twelve months based on 15 studies [11,23-27,29,30,33,34,37,41,44-46].

NNS to identify one eligible AUD patient was seven (2-32) based on 17 studies [11,19-22,26-30,35,37,39-41,44,47] and NNS to get one eligible AUD patient to accept intervention was ten (4-70) based on 18 studies [11,19-22,26-30,35,37,39-41,44,46,47].

#### Surgical patients

In two [12,31] of five studies the total patient population was given (529 and 3,783 patients respectively) as well as the number of screened patients. The screening acceptance rate in those two studies was 65% (47-83%); see figure 2.

The number of eligible AUD patients and number of patients accepting intervention was reported in all five studies; the acceptance rate for intervention among the eligible patients was 99% (54-100%); see figure 3.

The adherence rate after one month was 83% in the only study [13] that reported this number. The adherence rate after three months was 73% (53-92%) based on two studies [31,43], after six months 85% (82-88%) based on two studies [12,32] and 75% (69-81%) after twelve months based on two studies [31,43].

NNS to identify one eligible AUD patient was nine (3-15) based on two studies [12,31], and NNS to get one eligible AUD patient to accept intervention was 15 (3-28) based on the same two studies.

#### Subgroup analyses

The results showed that neither type of study, type of intervention nor validation of alcohol consumption affected the acceptance and adherence rates. Furthermore, the adherence rates were not affected by patients' group allocation or type of follow-up.

## Discussion

This review showed high acceptance rates for alcohol screening and intervention as well as adherence to intervention among emergency and surgical patients with AUDs. Four in five ED patients accepted alcohol screening compared to two in three surgical patients, whereas the intervention acceptance rate was to two out of three in ED patients compared to almost 100% among surgical patients. Though, as only a minority of the studies was conducted among surgical patients, no conclusions can be made regarding possible differences in emergency and surgical patients' acceptance of alcohol screening and interventions. Adherence to the alcohol intervention programs was above 60% for up to twelve months in both ED and surgical patients. Overall, the numbers needed to screen to identify one eligible AUD patient and to get one eligible patient to accept intervention varied from a few up to 70 patients.

Acceptance of alcohol screening among AUD patients in intervention trials was not reduced compared to studies performing screening exclusively [49-52], despite the fact that the consequences of being identified with an AUD in an intervention trial can be more comprehensive regarding study participation and possible changes in alcohol consumption.

The NNS are important when planning future interventions. Here the NNS to identify one AUD patient is not different from that described in primary care [53]. However, the NNS for acceptance of alcohol intervention is up to four times higher in primary care. This indicates a larger potential for conducting alcohol interventions in hospital settings.

In addition, the acceptance and adherence rates are comparable to those in smoking cessation intervention studies conducted in similar patient groups [54-57].

We were not able to show that any of the study characteristics facilitated or hindered acceptance or adherence, but the rates were probably influenced by multiple other factors for example patients sex, age and lifestyle [58].

### Bias and limitations

The weaknesses of this review are closely related to the weaknesses of the individual studies. In general, the included studies were heterogeneous. The intervention and primary outcomes differed from study to study. Screening methods used for detecting AUDs varied notably between the studies from blood tests to interviews and different questionnaires. The included questionnaires mainly focus on alcohol abuse and dependency and have mostly been developed in non-surgical settings [10]. These questionnaires may therefore not be useful for detecting a current hazardous alcohol intake in surgical patients, which is the most clinical relevant outcome [59]. Also, in emergency departments there does not appear to be a gold standard tool for screening for AUDs [60].

Moreover, the studies used different questionnaires and in the same questionnaires different cut-of points were sometimes applied. As a result a patient identified with AUD in one study would not necessarily be identified or included in another study. This could affect the NNS. In addition, differences in other inclusion and exclusion criteria between the studies could also influence the NNS.

Many studies had not reported the total patient population and number of patients accepting screening and alcohol intervention. Also, numbers of patients available for follow-up were not given in a few studies. Missing data from several studies may have affected the acceptance and adherence rates as well as the value of the subgroup analyses, where data in many cases was limited. Furthermore, as almost all studies described randomized trials and brief interventions comparisons among the few studies having other characteristics may not be valid.

### Generalization

No comparable systematic or narrative reviews were found. Other studies on patient opinions and experiences found that patients in EDs were positive towards alcohol screening including blood tests [61,62]. Also, in interviews with AUD patients following intervention they described the ED as an appropriate setting [63]. A recent study among acute surgical patients with AUDs sustained that alcohol intervention is relevant in relation to surgery [64].

Though generalization of the results should be considered carefully, the ED patients and patients undergoing surgery do not seem to form a major barrier for introducing alcohol screening and intervention programs. The high acceptance and adherence rate may also reflect staff effort and compliance to research protocol. Compared to project staff, the clinical staff may experience other barriers for alcohol screening and intervention such as missing knowledge and training as well as lack of time, appropriateness of setting and implementation into daily routines [65-67]. These barriers may, however, be overcome by prioritizing the area through professional teaching and training as well as using lessons learned regarding appropriate setting and resources.

The clinical routines differ from study settings in several ways. In general, projects and studies benefit from a very professional approach from trained and experienced staff, who are also highly dedicated to screen patients, intervene and follow-up. Study settings have also been tailored to meet the requirements for completing the project parts. In this way, several of the barriers above are overcome.

### Perspectives

A background WHO-paper concluded that there are no technical barriers for handling alcohol intervention as well as other hospital-based health promoting activities in the DRG-system [68]. Furthermore, a simple model for documentation of patient need for alcohol intervention and the related health promotion activity was shown to be useful, applicable and understandable when evaluated internationally in clinical settings [69]. These international tools are an integrated part of WHO standards and they all fit into the hospitals' quality management [70].

## Conclusions

In conclusion, this review showed that most emergency and surgical patients with AUDs accept participation in alcohol screening and interventions and complete the intervention program.

## Abbreviations

AUD: Alcohol Use Disorder; ED: Emergency Department; NNS: Numbers needed to screen

## Competing interests

The authors declare that they have no competing interests.

## Authors' contributions

Two authors (BP and KO) independently screened titles and abstracts from the identified papers, and articles with clearly irrelevant content were excluded. The same two authors independently assessed all other articles according to the inclusion criteria. A third author (LE) performed the additional search in reference lists, databases etc. Any disagreement was solved by consensus involving all authors (BP, KO, LE and HT). All authors read and approved the final manuscript.

## Pre-publication history

The pre-publication history for this paper can be accessed here:

http://www.biomedcentral.com/1471-2482/11/26/prepub

## Supplementary Material

Additional file 1 **Table S2 - Characteristics of 33 included studies involving ED or surgical patients**. Click here for file
