# Supplementary material for: Will emergency and surgical patients participate in and complete alcohol interventions? A systematic review
Source: BMC Surg. 2011 Sep 23;11:26. doi: 10.1186/1471-2482-11-26 (PMC3189859; doi:10.1186/1471-2482-11-26)
Supplement: Additional file 1 — Table S2 - Characteristics of 33 included studies involving ED or surgical patients. [file 1471-2482-11-26-S1.DOC]

## Table S2 - Characteristics of 33 included studies involving ED or surgical patients

| **Study** | Design | **Definition of AUD** | **Identification of AUD** | **Total population** | Number accepting screening | **Number eligible AUD patients** | **Number accepting intervention** | **Type of intervention and follow-up (months)** |
| --- | --- | --- | --- | --- | --- | --- | --- | --- |
| Emergency department patients | | | | | | | | |
| Antii-Poika et al, 1988 | RCT | Heavy drinking | MAST^1^ | Missing | 449 | Missing | 120 | Intensive; Attendance (6) |
| Bazargan-Hejazi et al, 2005 | RCT | At-risk drinking | CAGE^3^ | 1,058 | 1,036 | 488 | 295 | Brief; Attendance (3) |
| Blow et al, 2006 | RCT | At-risk drinking | Quantity x Frequency | 6,047 | 5,540 | 649 | 575 | Brief; Phone  (3 + 12) |
| Cherpitel et al, 2009 | RCT | At-risk + dependent drinking | RAPS^4^ + Quantity x Frequency | 2,815 | 1,913 | 494 | 446 | Brief; Phone  (3) |
| Coulton et al, 2009 | RCT | Hazardous drinking | SISPS-PAT^5^ + M-SASQ^6^ + FAST^7^ | Missing | 7,830 | Missing | 1,179 | Brief; Attendance (6 + 12) |
| Crawford et al, 2004 | RCT | Alcohol misuse | PAT^8^ | Missing | 5,240 | 657 | 599 | Brief; Phone or attendance (6 + 12) |
| Crawford et al, 2010 | RCT | Alcohol misuse | PAT^8^ | Missing | 914 | 187 | 103 | Brief; Phone or attendance (3 + 6) |
| Daeppen et al, 2007 | RCT | Hazardous drinking | Quantity x Frequency | 8,439 | 5,136 | 1,247 | 987 | Brief; Phone  (12) |
| Dauer et al, 2006 | RCT | Alcohol positive traffic casualties | BAC^9^ | 1,106 | 948 | 126 | 85 | Brief; Unknown  (3, 6 + 12) |
| Dent et al, 2008 | RCT | High-risk alcohol use | PAT^8^ | 32,965 | 10,274 | 1,043 | 468 | Brief; Phone  (1 + 3) |
| D’Onofrio et al, 2008 | RCT | Hazardous + harmful drinking | NIAAA^2^ | Missing | 16,182 | 571 | 500 | Brief; Phone  (6 + 12) |
| Forsberg et al, 2000 | RCT | Alcohol problems | Mm-Mast^10^ | 697 | 563 | 186 | 186 | Brief; Phone or attendance (6 + 12) |
| Gentilello et al, 1999 | RCT | Alcohol problems | BAC^9^+ SMAST^11^ + GGT^12^ | 5,640 | 2,524 | 1,153 | 762 | Brief; Attendance (6 + 12) |
| Magill et al, 2009 | RCT | Alcohol problems | BAC^9^ + AUDIT^13^ | Missing | Missing | Missing | 215 | Brief; Unknown  (6 + 12) |
| Mello et al, 2005 | RCT | Hazardous + harmful drinking | BAC^9^ + AUDIT^13^ | Missing | 3,756 | 921 | 539 | Brief; Attendance, phone or mail  (12) |
| Mello et al, 2008 | RCT | High-risk alcohol use | NIAAA^2^ | 6,335 | 6,086 | 1,329 | 285 | Brief; Phone  (3) |
| Monti et al, 1999 | RCT | Alcohol positive | BAC^9^ + AUDIT^13^ | Missing | Missing | Missing | 94 | Brief; Phone (3) & Attendance (6) |
| Monti et al, 2007 | RCT | Problem drinking | BAC^9^ + AUDIT^13^ | 5,607 | 3,125 | 627 | 198 | Brief; Unknown  (6 + 12) |
| Neumann et al, 2006 | RCT | At-risk drinking | AUDIT^13^ | 8,620 | 3,026 | 1,183 | 1,139 | Brief; Phone  (6 + 12) |
| Rodriguez et al, 2003 | RCT | Alcohol-related traffic casualties | Alcohol-saliva test | 709 | 696 | 93 | 66 | Brief; Phone  (3, 6 + 12) |
| Rodriguez et al, 2005 | RCT | Alcohol positive traffic casualties | BAC^9^ | 1,106 | 948 | 126 | 85 | Brief; Phone  (3, 6 + 12) |
| Roudsari et al, 2009 | RCT | Alcohol overconsumption | BAC^9^ + CAGE^3^ + NIAAA^2^ | 9,448 | 5,735 | 2,366 | 1,493 | Brief; Phone  (6 + 12) |
| Schermer et al, 2006 | RCT | Alcohol disorders | BAC^9^ | Missing | 1,125 | 157 | 126 | Brief; No follow-up |
| Soderstrom et al, 2007 | RCT | At-rsik drinking | AUDIT^13^ + CAGE^3^ | 5,670 | 5,453 | 624 | 497 | Brief; Phone  (6 + 12) |
| Sommers et al, 2006 | RCT | Alcohol-related vechicular injury | BAC^9^ | Missing | 4,618 | 481 | 187 | Brief; Phone  (12) |
| SBIRT research, 2007 | CCT | Unhealthy alohol use | NIAAA^2^ | 8,909 | 7,751 | 1,509 | 1,132 | Brief; Phone  (3) |
| Stein et al, 2009 | RCT | Hazardous drinking | BAC^9^ + AUDIT^13^ | 3,756 | Missing | 42 | 42 | Brief; Attendance, phone or mail  (3 + 12) |
| Trinks et al, 2010 | RCT | Risk Drinking | Quantity x Frequency | 3,848 | 1.570 | 560 | 145 | Brief; Mail  (6) |

| Surgical patients | | | | | | | | |
| --- | --- | --- | --- | --- | --- | --- | --- | --- |
| Goodall et al, 2008 | RCT | Hazardous drinking | AUDIT^13^ | 529 | 249 | 195 | 194 | Brief; Unknown (3 & 12) |
| Holloway et al, 2007 | RCT | Heavy drinking | Quantity x Frequency | Missing | Missing  Missing/56/56 | 56 | 56 | Brief; Attendance (6) |
| Shourie et al, 2007 | CCT | Excessive alcohol consumption | WHO Tri-level method | 3,783 | 3,139 | 250 | 136 | Brief; Attendance (6) |
| Smith et al, 2003 | RCT | Alcohol-related face injury | Drinking prior to injury | Missing | 219 | 166 | 151 | Brief; Attendance, phone or mail (3 & 12) |
| Tønnesen et al, 1999 | RCT | Alcohol misusers | Quantity x Frequency | Missing | Missing | 42 | 42 | Intensive; Attendance (1) |
| ^1^ Michigan Alcoholism Screening Test, ^2^ National Institute of Alcohol Abuse and Alcoholism’s low-risk limits, ^3^ Cut down, Annoyed, Guilty, Eye-Opener, ^4^ Positive Rapid Alcohol Problems Screen, ^5^ Modified Paddington Alcohol Test, ^6^ Single Alcohol Screening Questionnaire, ^7^ Fast Alcohol Screening Test, ^8^ Paddington Alcohol Test, ^9^ Blood Alcohol Concentration, ^10^ Malmo-modified Michigan Alcoholism Screening Test, ^11^ Short Michigan Alcoholism Screening Test, ^12^ Serum gamma glutamyl transpeptidase, ^13^ Alcohol Use Disorder Identification Test | | | | | | | | |
